# Malocclusion Generates Anxiety-Like Behavior Through a Putative Lateral Habenula–Mesencephalic Trigeminal Nucleus Pathway

**DOI:** 10.3389/fnmol.2019.00174

**Published:** 2019-07-30

**Authors:** Xin Liu, Kai-Xiang Zhou, Nan-Nan Yin, Chun-Kui Zhang, Ming-Hong Shi, Hong-Yun Zhang, Dong-Mei Wang, Zi-Jun Xu, Jing-Dong Zhang, Jin-Lian Li, Mei-Qing Wang

**Affiliations:** ^1^State Key Laboratory of Military Stomatology, Department of Oral Anatomy and Physiology, National Clinical Research Center for Oral Diseases & Shaanxi International Joint Research Center for Oral Diseases, School of Stomatology, The Fourth Military Medical University, Xi’an, China; ^2^Department of Stomatology, The 960th Hospital of People’s Liberation Army, Jinan, China; ^3^Department of Anatomy, Histology and Embryology and K.K. Leung Brain Research Center, The Fourth Military Medical University, Xi’an, China; ^4^Department of Oral Medicine, School of Stomatology, Xinxiang Medical University, Xinxiang, China; ^5^School of Clinical Medicine, University of South China, Hengyang, China; ^6^Department of Anesthesiology, University of Cincinnati Medical College of Medicine, Cincinnati, OH, United States

**Keywords:** anxiety, lateral habenula, trigeminal mesencephalic nucleus, unilateral anterior cross bite, vesicular glutamate transporter-2

## Abstract

Malocclusion is an important risk factor for temporomandibular disorder (TMD), a series of disorders characterized by dysfunction in the orofacial region involving the temporomandibular joint (TMJ) and jaw muscles. We recently showed that experimental unilateral anterior crossbite (UAC) produced masseter hyperactivity through a circuit involving the periodontal proprioception, trigeminal mesencephalic nucleus (Vme), and trigeminal motor nucleus (Vmo). Anxiety is a common complication in patients with TMD. The lateral habenula (LHb) is involved in emotional modulation and has direct projections to the Vme. Therefore, the present research examined whether UAC facilitates excitatory input from the LHb to the Vme and, subsequently, anxiety-like behaviors in rats. The LHb activation was evaluated by the electrophysiological recording, assessment of vesicular glutamate transporter-2 (VGLUT2) mRNA expression, and measurement of anxiety-like behaviors. The effects of LHb activity on Vme were evaluated by electrophysiological recording from Vme neurons and local changes in VGLUT2 protein density. UAC produced anxiety in modeled rats and increased neuronal activity in the LHb. VGLUT2 mRNA expression was also increased in the LHb. Further, VGLUT2-positive boutons were observed in close apposite upon parvalbumin (PV)-labeled Vme neurons. VGLUT2 protein expression was also increased in the Vme. Significantly, injection of VGLUT2-targeted shRNA into the LHb reduced the expression of VGLUT2 protein in the Vme, attenuated UAC-associated anxiety-like behaviors, and attenuated electrophysiological changes in the Vme neurons. In conclusion, we show that UAC activates the LHb neurons as well as the periodontal proprioceptive pathway to provide excitatory input to the Vme and produce anxiety in rats. These findings provide a rationale for suppressing activity of the LHb to attenuate both the physical and psychological effects of TMD.

## Introduction

Masseter hyperactivity is often observed in temporomandibular disorder (TMD), which is a collective term for a heterogeneous array of psychosocial and physiological disorders that is more prevalent in females (Klasser et al., 2018). Based on both cross-sectional studies (Wang et al., 2009) and animal experiments (Huang et al., 2002; Henderson et al., 2015; Zhang et al., 2016), aberrant dental occlusion plays an adverse role in TMD. Occlusion determines muscular performance *via* periodontal–muscular feedback mechanisms that are modified by contact-mediated loading (Wang and Mehta, 2013). Contact changes, such as a posterior crossbite, are found in 8%–22% of humans (Agostino et al., 2014) and have an impact on chewing movements (Piancino et al., 2006, 2012). However, in rodents such as rats, the anterior teeth or incisors are more frequently used in chewing. Based on the chewing pattern of rodents, we developed a unilateral anterior crossbite (UAC) rat model in which osteoarthritic lesions of the temporomandibular joint (TMJ) are induced (Zhang et al., 2018). As we proposed in our recent publication, UAC elicited hyperactivity of jaw closing muscles, probably through a circuit of periodontal mesencephalic trigeminal afferents (Vme) to trigeminal motor neurons (Vmo; Liu et al., 2017). A similar impact of the Vme on multiple orofacial motor nuclei in the brain stem has also been reported (Liu et al., 2018). Therefore, the UAC model could be used in explorations of Vme-related problems, which are worthy of investigation.

Psychological stress is also a well-documented risk factor for TMD (Pallegama et al., 2005). Laskin (1969) was the first scholar to define a psychological subset of TMD, which was termed myofascial pain dysfunction syndrome. He assumed that muscle fatigue is the most common cause of TMD. A previous report suggested that anxiety is a major risk factor for TMD among university (Casanova-Rosado et al., 2006) and pre-university students (de Lucena et al., 2012). Consistent with these findings, patients with TMD tend to be more anxious and/or depressed than asymptomatic control subjects (Gameiro et al., 2006). In addition, TMD symptoms manifest more frequently when patients are suffering from stress (de Leeuw et al., 1994; Suvinen et al., 1997). Recently, a psychological assessment was recommended as a criterion in the clinical examination and study of TMD (Ohrbach and Dworkin, 2016). However, how or through which brain network anxiety or stress can exert an effect on occlusion and consequently be involved in the development of TMD remains unclear.

The habenula is a pair of small nuclei located above the posterior end of the thalamus, close to the midline. The habenula is divided into the medial habenula and lateral habenula (LHb; Paxinos and Watson, 1998). The LHb plays a well-characterized negative regulatory role in monoaminergic systems in the central nervous system (Wang and Aghajanian, 1977; Christoph et al., 1986; Matsumoto and Hikosaka, 2007). Consequently, the LHb is involved in a set of depressive symptoms (Yang et al., 2008; Hikosaka, 2010; Li et al., 2013) and sleep disturbances (Aizawa et al., 2013). LHb responses to stress were initially unveiled by the observation that LHb neurons are activated by a variety of stressors and aversive/unpleasant stimuli (Jacinto et al., 2017). Recently, Ohara et al. (2016) reported a direct projection from the LHb to the Vme using anterograde and retrograde tract tracing. The neurons that directly project to the Vme are restricted in the LHb but not in the vicinity (Ohara et al., 2016). Most LHb efferent neurons are glutamatergic (Fremeau et al., 2001), and they use vesicular glutamate transporter 2 (VGLUT2) to fill their presynaptic vesicles, while glutamatergic Vme neurons use VGLUT1 to fill their projections to the Vmo (Pang et al., 2006). Thus, the increase in VGLUT2 proteins in Vme areas implies one possibility of enhancement of LHb afferent signals if this protein increase is paralleled by VGLUT2 mRNA upregulation in the LHb. If so, injection of VGLUT2 shRNA into the LHb would reverse VGLUT2 mRNA expression in the LHb and reduce protein levels in the Vme.

In light of these previous studies and our own observations, we hypothesize that UAC, as an unpleasant external stimulation, generates anxiety mediated by the LHb. Enhanced LHb neuronal activity may also facilitate Vme neuronal activity through a direct projection (Ohara et al., 2016). Confirmation of this prediction will provide new insights into the psychosocial and physiological effects of dental problems in view of their etiology and possible therapeutic strategies.

## Materials and Methods

A total of 230 Sprague–Dawley rats (130–160 g; 6 weeks of age; more than half were female) were provided by the Animal Center of the Fourth Military Medical University and divided into three major groups. All experimental procedures were conducted in accordance with the Principles of Laboratory Animal Care and approved by the University Ethics Committee and performed as per institutional guidelines. Efforts were made to minimize the number of animals used.

### UAC Modeling

UAC surgery was performed on 6-week-old rats. Rats were anesthetized with sodium pentobarbital (40 mg/kg, intraperitoneal injection, i.p.) and administered atropine sulfate (0.5 mg/kg, i.p.) to reduce tracheal secretions. There are two pairs of incisors in each rat, and the maxillary incisors normally bite on the labial side of the mandibular incisors. Metal tubes were affixed to the left-side incisors to establish UAC as we previously described (Lu et al., 2015; Liu et al., 2017). Briefly, a section of a metal tube cut from a pinhead (length, 2.5 mm; inside diameter, 3 mm) was affixed to the left maxillary incisor and a curved section of metal tube (length, 4.5 mm; inside diameter, 3.5 mm) was affixed to the left mandibular incisor. The tubes were carefully bonded with zinc phosphate cement and checked every 2 days. No prostheses were found detached during the experimental period. In the control UAC group, rats received the same procedure without fixation of the metal tubes. All animals were fed with cylindrically shaped pressed food pellets.

#### Verification of UAC-Induced Anxiety and Relevant Behavior, Neurophysiology, and Biochemistry Changes

The first group of rats was examined to determine whether UAC elicited anxiety-like behavior and whether the LHb responded to UAC with any anxiety-associated changes, such as enhanced neuron activity and/or increases in neurobioactive substances. Thus, we divided this group into three subgroups, termed groups 1.1, 1.2, and 1.3, in which anxiety-like behavior (group 1.1), LHb neurophysiological properties (group 1.2), and neurobioactive substance expression levels (group 1.3) were tested.

For group 1.1, 12 rats were used for open-field testing, and an additional 12 rats were used for elevated plus-maze testing at baseline. Animals in both testing groups were randomly assigned to sham surgery as a control (*n* = 6) or UAC surgery (*n* = 6). Behavioral testing was performed at six postoperative time points (3 h, 1 week, 2 weeks, 4 weeks, 8 weeks, and 12 weeks), and the rats were euthanized at the end of the 12 weeks. The open-field test was performed as follows: an open-field chamber (RD1412-OF, Shanghai Mobile Datum Corporation, Shanghai, China) consisting of a 100 cm × 100 cm × 80 cm Plexiglas box was settled in a temperature-controlled room and illuminated by a single fluorescent light suspended over the chamber. The activity of the rat was monitored for 15 min by an automated analysis system (Shanghai Mobile Datum Information Technology). The total distance moved and the percentage of time spent in the center area (center time%) were used as parameters for evaluating anxiety levels by off-line analysis. All animals were habituated to the testing room for 30 min before the test. The elevated plus-maze test was conducted as follows: an elevated plus-maze apparatus (RD1208-EP, Shanghai Mobile Datum Corporation, Shanghai, China), which comprised two open arms (OAs; 50 cm × 10 cm) and two enclosed arms (CAs; 50 cm × 10 cm × 40 cm) that extended from a common central platform (10 cm × 10 cm). The plus-shaped platform was placed 70 cm above the floor in a temperature-controlled room. For testing, the rats were placed in the central square (10 cm × 10 cm) among the closed and opened arms and allowed to explore the elevated plus-maze for 5 min. The numbers of OA and CA entries and the times spent in the OAs and CAs were recorded by an automated analyzing system (Shanghai Mobile Datum Information Technology). The test relies on the animal’s natural fear of open spaces, and the percent of time spent in OAs (OA time percentage) and the percent of OA entries (OA entries percentage) are considered measurements of general anxiety/depression levels. OA time percentage = OA time/(OA time + CA time). OA entries percentage = OA entries/(OA entries + CA entries) (Tordera et al., 2007; Pohorecky, 2008).

In group 1.2, 12 LHb neurons from three UAC rats and 8 LHb neurons from three control rats were targeted intracellularly to record their neurophysiological properties using both current and voltage clamp techniques. The electrophysiological experiment was conducted in the 8th week after the UAC or sham surgery was performed. Brain slices containing the LHb were prepared as follows. The rats were rapidly decapitated, and brains were immediately removed and placed in carbogen (95% O_2_, 5% CO_2_)-bubbled ice-cold sucrose artificial cerebrospinal fluid (ACSF, 248 mM sucrose substituted for NaCl) for 30 min. Then, 200- to 220-μm coronal slices of the LHb were cut through the midbrain on a Leica vibratome (Leica VT 1200s, Heidelberger, Nussloch, Germany) in ice-cold sucrose ACSF according to the coordinates of the LHb based on the Rat Brain Atlas (Paxinos and Watson, 1998) that were 3.6 ± 0.05 mm caudal to Bregma, 0.8 ± 0.05 mm lateral to the midline, and 5.4 ± 0.05 mm deep from the brain surface. The slices were quickly transferred into cold oxygenated regular ACSF (124 mM NaCl, 2.5 mM KCl, 2 mM MgSO_4_ + 7H_2_O, 2 mM CaCl_2_, 1 mM NaH_2_PO_4_, 25 mM NaHCO_3_, 25 mM glucose, 1 mM ascorbate, and 3.0 mM pyruvate) and recovered from processing shock for 1 h at room temperature (RT) before recording. In current clamp mode, the firing patterns of LHb neurons in UAC and control rats were compared by analyzing the trains of action potentials (APs) evoked by intracellular injection of 0-, 25-, 50-, 75-, 100-, and 125-pA depolarizing currents for 500 ms. In voltage clamp mode, currents were recorded at a holding potential of −70 mV (intra-electrode solution containing 130 mM potassium gluconate, 5 mM NaCl, 15 mM KCl, 0.4 mM EGTA, 10 mM HEPES, 4 mM Mg-ATP, and 0.2 mM Tris–GTP; pH 7.2–7.4; osmolality 290–300 mOsm), and spontaneous excitatory postsynaptic currents (sEPSCs) were recorded 10–15 min after establishing whole-cell access, and the current reached a steady state. All signals were recorded using a Multi Clamp 700B Amplifier (Axon Instruments, Forster City, CA, USA) that connected to a Dell computer installed with pClAMP 10.2 software (Axon Instruments).

In group 1.3, 72 rats were used to detect expression changes of VGLUT2 in the LHb as a potential response to UAC-induced anxiety since VGLUT2 is a vehicle used to transport glutamic acid into synaptic vesicles in the LHb efferent neuronal boutons, and its level represents the degree of activity of the LHb neurons as mentioned in the “Introduction” section. Rats were randomly assigned to sham surgery as controls (*n* = 36) or UAC surgery (*n* = 36) and were euthanized at six postoperative time points (3 h, 1 week, 2 weeks, 4 weeks, 8 weeks, and 12 weeks; *n* = 6 at each time point). Half of the rats sacrificed at each time point were used for *in situ* hybridization, and the remaining rats were used for real-time polymerase chain reaction (PCR). In the *in situ* hybridization test, a VGLUT2 complementary DNA fragment was cloned into the vector pBluescriptII SK+ (Stratagene, La Jolla, CA, USA). Sense and antisense single-stranded RNA probes were synthesized with a digoxigenin (DIG) labeling kit (Roche Diagnostics, Basel, Switzerland) using the plasmids as a template. Frozen sections containing the LHb were cut at 30-μm thickness and directly mounted on anti-RNAase-treated slides. The sections were hybridized with DIG-labeled sense and antisense RNA probes (0.5 μg/ml) against VGLUT2 mRNA for 16–24 h at 60°C. After two washes in 2× saline sodium citrate (SSC; 0.15 M NaCl and 0.015 M sodium citrate, pH 7.0) for 20 min each at 60°C, the sections were incubated with RNase A (10 μg/ml) in a mixture of 0.5 M NaCl, 0.01 M Tris–HCl (pH 8.0), and 1 mM ethylenediaminetetraacetic acid for 30 min at 37°C and then washed in 0.2× SSC containing 0.1% (w/v) *N*-lauroylsarcosine for 20 min at 37°C. Subsequently, the sections were incubated with an alkaline phosphatase-conjugated anti-DIG antibody (diluted 1:2,000) and Fab fragment (1093274; Roche Diagnostics, Basel, Switzerland) in 0.1 M Tris–HCl (pH 7.5) buffered 0.9% (w/v) saline containing 1% blocking reagent. The next day, the sections were washed with TNT containing 0.15 M NaCl, 0.1 M Tris–HCl (pH 7.5), and 0.05% (v/v) Tween 20 and incubated for 3 h with fluorescein isothiocyanate (FITC)-avidin (1:1,000; Millipore, Bedford, MA, USA). Slides were sealed with Vectashield (Vector, Burlingame, CA, USA) and observed under a confocal laser scanning microscope (FV1000; Olympus, Tokyo, Japan). Digital images were captured and processed using FV10-ASW 1.6 software (Olympus).

In the real-time PCR test, brain slices containing the LHb were cut in the same way described for electrophysiological preparation except RNAase inhibitor was added to the ACSF. The LHb area was identified under an anatomic microscope based on the well-established Rat Brain Atlas (Paxinos and Watson, 1998) and was dissected out carefully. RNA was isolated from LHb tissue using TRIzol (Invitrogen, Carlsbad, CA, USA) and purified with an RNeasy Mini Kit (Qiagen, Valencia, CA, USA). Gene expression was analyzed using an Applied Biosystems 7500 Real-time PCR machine (Applied Biosystems, CA, USA). The primers for VGLUT2 were CCCGTCTACGCGATAATTGTT (forward) and GTCATGACAAGGTGAGGGACT (reverse). Target mRNA levels were normalized and are displayed as fold changes compared to those of the naïve control group.

#### Identification of the Neuronal Pathway Linking Anxiety to Occlusion

The second group of rats was used to confirm the involvement of a neuronal pathway from the LHb to the Vme in anxiety-associated jaw muscle hyperactivity, as direct projection from the LHb to the Vme was recently reported (Ohara et al., 2016). In this group, two subgroups, groups 2.1 and 2.2, were created in which neuronal tract tracing of the LHb–Vme pathway was combined with immunolabeling of both VGLUT2 terminals and Vme neurons, as well as morphometric and Western blot analysis of VGLUT2 protein levels in the Vme.

Group 2.1 contained six rats for neuronal tract tracing using biotinylated dextran amine (BDA; 10,000 MW, Molecular Probes, Eugene, OR, USA) as a tracer. Animals were anesthetized with sodium pentobarbital (40 mg/kg, i.p.) plus atropine sulfate (0.5 mg/kg, i.p.) and were placed into a stereotaxic frame after the pain withdrawal reflex disappeared. The left parietal bone was partially removed, and a glass micropipette (10–15 μm thick tip) filled with 4% BDA in saline was advanced 5.4 ± 0.05 mm aiming at the LHb according to the Rat Brain Atlas (Paxinos and Watson, 1998), with the coordinates of 3.6 ± 0.05 mm caudal to Bregma and 0.8 ± 0.05 mm lateral to the midline. BDA was delivered ionophoretically (positive pulses, 2 μA, 300 ms, 2 Hz) for 20–30 min, and then the incision was closed by suturing. An antibiotic (cefotiam hydrochloride, 66 mg/kg, i.p.) and analgesic (flurbiprofen axetil, 3.3 mg/kg, i.p.) were administered before allowing the rats to recover. The rats survived without bleeding or inflammation for 5 days and were then euthanized by an overdose of sodium pentobarbital (120 mg/kg, i.p.) and transcardially perfused with saline followed by 4% (w/v) paraformaldehyde in 0.1 M phosphate buffer (PB; pH 7.3).

Immunofluorescent visualization of the BDA (injection site and terminals), anti-VGLUT2, and Vme neuronal somas was carried out by incubating the sections with a mixture of guinea pig anti-VGLUT2 antibody (1:500; Synaptic System, Goettingen, Germany) and mouse anti-parvalbumin (PV) antibody (1:1,000; Millipore) for 16–18 h. Then, the sections were incubated with a cocktail of FITC-avidin (1:1,000; Millipore), Alexa 594-conjugated donkey anti-guinea pig immunoglobulin G (IgG, 1:500; Millipore), and Alexa 647-conjugated donkey anti-mouse IgG (1:500; Millipore) for 4 h at RT. Control staining was performed by omission of primary antibodies. Slides were sealed with Vectashield (Vector) and observed under a confocal laser scanning microscope (FV1000). Digital images were captured and processed using FV10-ASW 1.6 software (Olympus).

In group 2.2, 72 rats were randomly assigned to receive sham surgery (*n* = 36) or UAC surgery (*n* = 36) and were euthanized at six postoperative time points (3 h, 1 week, 2 weeks, 4 weeks, 8 weeks, and 12 weeks; *n* = 6 at each time point). Animals in this group were used to detect VGLUT2 protein levels in the Vme; 36 rats were used for immunofluorescent visualization, and the remaining rats were used for Western blotting. For microscopic visualization of VGLUT2 density in the Vme and VGLUT2-positive boutons, sections were incubated at RT with a mixture of guinea pig anti-VGLUT2 antibody (1:500; Synaptic System) and mouse anti-PV antibody (1:1,000; Millipore) for 16–18 h. The sections were then incubated with a mixture of Alexa 594-conjugated donkey anti-guinea pig IgG (1:500; Millipore) and Alexa 647-conjugated donkey anti-mouse IgG (1:500; Millipore) for 4 h at RT. Control immunostaining was performed by omission of the primary antibody. Slides were sealed with Vectashield (Vector) and observed under a confocal laser scanning microscope (FV1000; Olympus). Digital images were captured and processed using FV10-ASW 1.6 software (Olympus). For Western blotting, rats were initially processed in the same way described for electrophysiological preparation. The Vme is located at the ventrolateral end of the periaqueductal gray matter of the midbrain and at the lateral end of the gray matter of the floor of the fourth ventricle. At the level of the pons, the Vme is located just lateral to the locus coeruleus. The coordinates of Vme are 0.4–1.0 mm posterior to the interaural line, 1.4–1.6 mm lateral to the midline, and 6.75–6.85 mm in depth from the brain surface based on the Rat Brain Atlas (Paxinos and Watson, 1998). The Vme area was localized at the coronal plane under an anatomic microscope (SZX7-1013, Olympus) and was dissected out carefully. Tissues were homogenized in extraction buffer (20 mM Tris–HCl, pH 7.4, 5 mM EDTA, 140 mM NaCl, 1% Triton X-100, 1 mM Na_3_VO_4_, 1 mM PMSF, 10 mM NaF, and 1 mg/ml aprotinin) at 4°C and were centrifuged at 12,000× *g* for 20 min at 4°C. The protein content in the supernatant was measured with a Bio-Rad Protein Assay kit (Bio-Rad, Hercules, CA, USA) and denatured at 95°C for 5 min with 5× SDS-loading buffer before separation with sodium dodecyl sulfate-polyacrylamide gel electrophoresis (SDS-PAGE) and transfer to an Immobilon-P membrane. Membranes were blocked with 5% non-fat milk for 2 h and incubated with primary antibodies against mouse anti-β-actin (1:2,000, Santa Cruz Biotechnology, CA, USA) and mouse anti-VGLUT2 (1:500; Millipore) overnight at 4°C. Next, blots developed using a horseradish peroxidase-conjugated secondary antibody and enhanced chemiluminescence (ECL). The immunolabeled band was detected by the ECL detection method (Amersham Pharmacia Biotech, Piscataway, NJ, USA) and exposure to film. The scanned images were quantified and analyzed with ImageJ software. Target protein levels were normalized against β-actin levels and expressed as fold changes relative to those of the naive control group.

#### Attenuation of Anxiety-Related Reactions by Intervention of the Aforementioned Neuronal Pathway

In the third group, 50 rats were used to explore whether downregulating VGLUT2 expression in the LHb would attenuate animal reactions to UAC-induced anxiety, such as anxiety-related behavior and an increase in neuronal activity downstream of the pathway. We injected VGLUT2 shRNA vectors into the LHb to downregulate VGLUT2 expression in the LHb. Then, we compared the animal’s behavior and VGLUT2 mRNA and protein levels in the LHb and Vme and Vme neuronal activity between the control vehicle, UAC vehicle, and UAC shRNA vector-injected rats. The control vehicle group indicates that the vehicle was injected into the LHb of naïve rats. Then, these rats were divided into three subgroups: group 3.1 (behavior), 3.2 (PCR and Western blotting), and 3.3 (electrophysiology).

##### VGLUT2 shRNA Vector Preparation

Six pairs of rat VGLUT2-specific short hairpin oligonucleotides were designed and cloned into pAAV-U6-RNAi-eGFP (Shanghai Taitool Bioscience Co. Limited, China). The pAAV-U6-RNAi-eGFP vector contained two Esp3I (Thermo Fisher Scientific) recognition sites downstream of the U6 promoter for shRNA adaption. The six resulting VGLUT2 shRNA vectors (containing sequences expressing enhanced green fluorescent protein, EGFP) were co-transfected with reporter vectors (containing sequences expressing VGLUT2 and mCherry) into HEK293 cells. The most potent VGLUT2 shRNA vector was selected by detecting the weakest expression of mCherry in cells.

##### Injection of the Vector and Vehicle

VGLUT2 shRNA-encoded adeno-associated viral (AAV) vectors (2 μl; 9.68 × 10^7^ viral particles/μl; Shanghai Taitool Bioscience Co. Limited, Shanghai, China) were administered to anesthetized animals in a stereotaxic frame with a setup similar to that used for the tracer injections. The VGLUT2 shRNA vector was injected into the LHb bilaterally at the 6th week after UAC or sham surgery had been performed. The injection was carried out for approximately 2 min, and the micropipette was held in place for an additional 2 min after completion. Control vehicle and UAC vehicle rats were injected in the same way without the vector.

In group 3.1, real-time PCR and Western blotting were carried out in the same way as described for group 2.2; similarly, statistical analysis and comparison of VGLUT2 mRNA in the LHb and protein level in the Vme was performed between control vehicle, UAC vehicle, and UAC shRNA vector injection rats.

In group 3.2, behavior tests were conducted in the same way as previously described, and statistical comparisons were made between control vehicle, UAC vehicle, and UAC shRNA vector injection rats.

In group 3.3, brain slice patches with both current and voltage clamps were applied to Vme neurons as previously described for group 1.2. In the current clamp mode, the firing patterns of Vme neurons between the control vehicle, UAC vehicle, and UAC shRNA vector injection tissues were compared by analyzing trains of APs evoked by intracellular injection of 0-, 10-, 20-,30-, 40-, 50-, 60-, 70-, and 80-pA depolarizing currents for 400 ms. In the voltage clamp mode, currents were recorded at a holding potential of −70 mV (intra-electrode solution containing 130 mM potassium gluconate, 5 mM NaCl, 15 mM KCl, 0.4 mM EGTA, 10 mM HEPES, 4 mM Mg-ATP, and 0.2 mM Tris–GTP; pH 7.2–7.4; osmolality 290–300 mOsm), and sEPSCs were recorded 10–15 min after establishing whole-cell access and the current reached a steady state. Spike firing rate and sEPSC frequency and amplitude were analyzed and compared between control vehicle, UAC vehicle, and UAC shRNA vector injection tissues.

### Statistical Analysis

Data acquisition and analysis were performed by a researcher blinded to the study group assignment using SPSS 16.0 (SPSS Inc., IL, USA). Data are expressed as the mean ± standard error of the mean (SEM). Two-way analysis of variance (ANOVA) with Bonferroni’s multiple comparison tests or one-way ANOVA with Tukey’s multiple comparison *post hoc* tests were used for between-group comparisons (for example, the analysis of Western blot data with group and time as the main effects). The threshold for statistical significance was *p* < 0.05 or *p* < 0.01, as specified.

## Results

### UAC Induced Anxiety-Like Behavior, Elevated LHb Neuronal Activity, and Upregulated VGLUT2 mRNA in LHb Neurons

**Figure 1:**
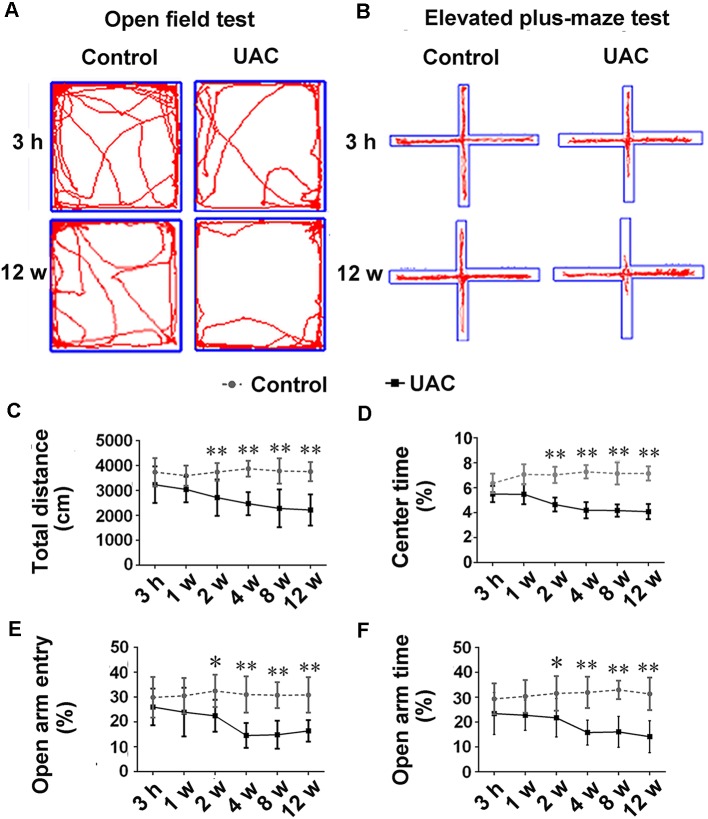
Effect of unilateral anterior crossbite (UAC) on anxiety-related behavioral indexes. **(A)** Rat movement trajectory in the open field test. **(B)** Rat motion trajectory in the elevated plus-maze test. **(C,D)** In the open field test, the total distance that rats reached **(C)** and the percentage of time spent in the center area **(D)** were significantly different between control and UAC-modeled ones (*p* < 0.05 at 2 weeks, *p* < 0.01 at 4–12 weeks). **(E,F)** Percentage of time that rats spent in open arms **(E)** and in the elevated plus-maze **(F)** were also significantly different between aforementioned two groups (*p* < 0.05 at 2 weeks, *p* < 0.01 at 4–12 weeks). *p < 0.05; **p < 0.01.

In group 1.1, representative motion trails in the open-field test and elevated plus-maze test are shown in Figures 1A,B, respectively. The open-field test showed that UAC rats traveled shorter distances (*p* < 0.05 at 2 weeks; *p* < 0.01 at 4, 8, and 12 weeks; Figure 1C) and spent less time in the center (*p* < 0.01 at 2, 4, 8, and 12 weeks; Figure 1D) than control rats did at 2 weeks after UAC or sham surgery. Consistently, the elevated plus-maze test results showed that UAC rats had a lower percentage of entries into OAs (*p* < 0.05 at 2 weeks; *p* < 0.01 at 4, 8, and 12 weeks; Figure 1E) and a lower percentage of time spent in the OAs (*p* < 0.05 at 2 weeks; *p* < 0.01 at 4, 8, and 12 weeks; Figure 1F) than control rats did at 2 weeks after surgery.

**Figure 2:**
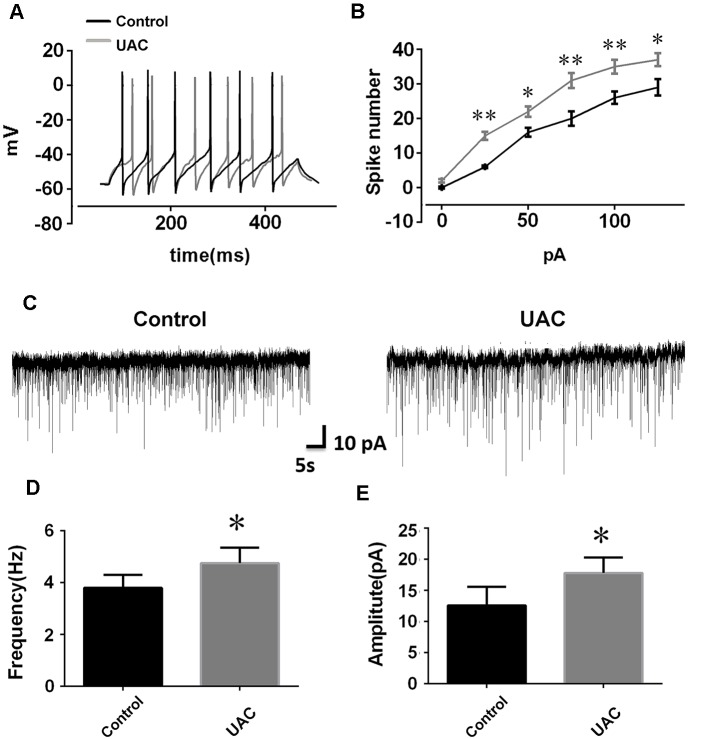
Effects of UAC on action potential (AP) frequency, and spontaneous excitatory postsynaptic currents (sEPSCs) in the lateral habenula (LHb) neurons. **(A)** Representative traces evoked by intracellular injection of 25-pA depolarizing currents on LHb neurons from control and UAC rats for 500 ms. **(B)** Average results showing that the number of APs in a train induced by the injection of step currents. UAC significantly increased the spike number when currents of 25 pA (*p* < 0.01), 50 pA (*p* < 0.05), 75 pA (*p* < 0.01), 100 pA (*p* < 0.01), and 125 pA (*p* < 0.05) were applied. **(C)** The representative traces of sEPSCs in LHb neurons recorded from control and UAC rats, respectively. **(D,E)** Both frequency **(D)** and amplitude **(E)** were significantly higher in UAC-modeled rats than that in control group (*p* < 0.05). *p < 0.05; **p < 0.01.

In group 1.2, the single cell recording under the current clamp revealed enhanced LHb neuronal activity in UAC rats. Representative traces evoked by intracellular injection of 25-pA depolarizing currents in LHb neurons from control and UAC rats are shown in Figure 2A. More averaged spikes were elicited in UAC rats than in control rats when currents of 25 pA (*p* < 0.01), 50 pA (*p* < 0.05), 75 pA (*p* < 0.01), 100 pA (*p* < 0.01), and 125 pA (*p* < 0.05) were applied (Figure 2B). The baseline frequency and amplitude of sEPSCs in UAC rats were significantly higher than those in control rats (*p* < 0.05, Figures 2C–E).

**Figure 3:**
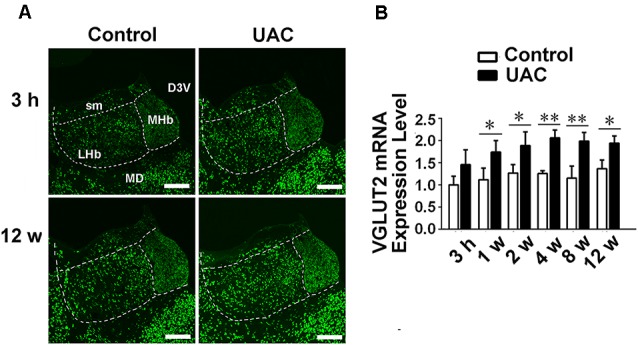
UAC upregulated vesicular glutamate transporter-2 (VGLUT2) mRNA expression in LHb neurons. **(A)** *In situ* hybridization immunofluorescence histochemical stains showed the hybridization signals in cell bodies of LHb neurons (in frame) at 3 h and 12 weeks in control and UAC groups. Scale bar = 200 μm. **(B)** Real-time polymerase chain reaction (PCR) analysis of VGLUT2 mRNA expression in LHb neurons showed that VGLUT2 mRNA levels, normalized to the GAPDH expression, were significantly changed as times of fold compared to those mRNA in the naive control group. *p < 0.05; **p < 0.01.

In group 1.3, the *in situ* hybridization approach revealed obvious VGLUT2 mRNA expression in LHb neurons occurring from 3 h to 12 weeks post-surgery, with more VGLUT2-positive neurons observed at the longer post-surgery time points, as shown in Figure 3A. Moreover, real-time PCR revealed higher VGLUT2 mRNA expression in the LHb neurons of UAC-treated rats than in those of control rats as early as 1 week post-surgery (*p* < 0.05 at 1, 2, and 12 weeks; *p* < 0.01 at 4 and 8 weeks; Figure 3B). These results showed that VGLUT2 mRNA expression alterations occurred earlier than animal behavior and LHb neuronal activity changes following UAC surgery.

### Anxiety Linked to Occlusion by a Neuronal Pathway from the LHb to Vme

**Figure 4:**
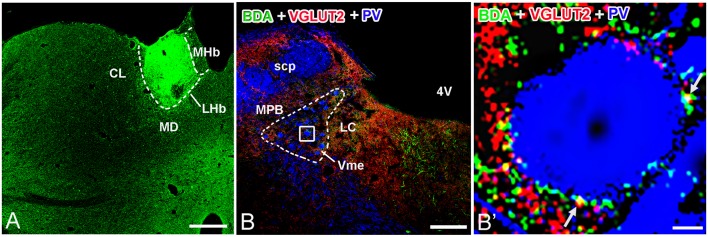
Biotinylated dextranamine (BDA), anti-VGLUT2, and parvalbumin (PV) triple labeling in trigeminal mesencephalic nucleus (Vme). **(A)** The BDA injection site was confined in the LHb. **(B)** Immunofluorescent triple labeling for BDA (green), VGLUT2 (red), and PV (blue) positive structures at a coronal section through the Vme in a rat that was injected with BDA into LHb. The framed area in **(B)** is magnified in **(B′)**. White arrows indicate axonal buttons dually labeled with VGLUT2 (red) and BDA (green) that are merged (yellow), and are in close apposition to PV-immunopositive Vme soma (blue). Scale bar = 300 μm in **(A)**; 200 μm in **(B)**; 10 μm in **(B′)**.

In group 2.1, neuronal tract tracing by injection of anterograde tracer to the LHb (Figure 4A) combined with immunostaining of VGLUT2 showed co-localization of anterograde tracer BDA and VGLUT2 proteins in LHb neuronal terminals (Figure 4B). Furthermore, using PV to stain Vme neuronal somas, we observed a large number of BDA and VGLUT2 double-labeled boutons that were close to PV-positive somas (Figure 4B′).

**Figure 5:**
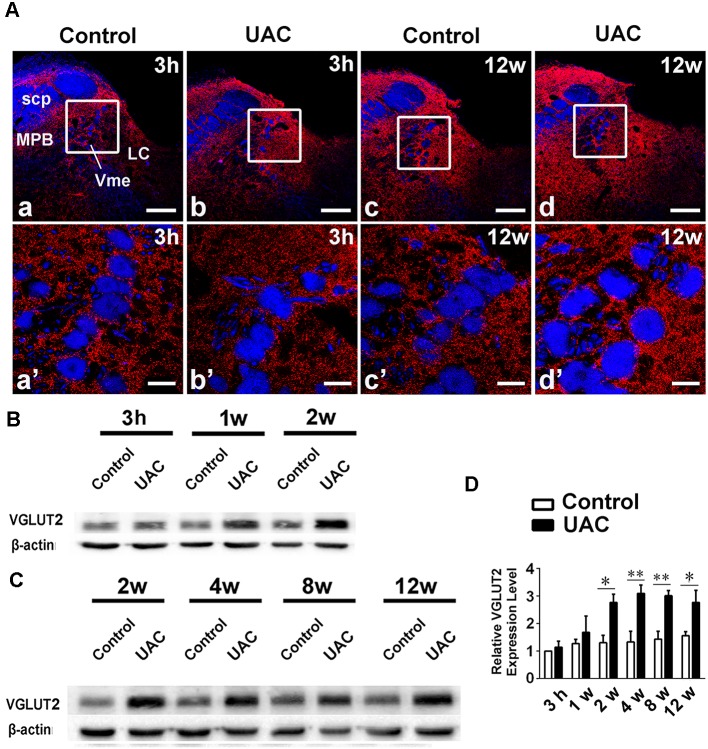
VGLUT2 protein level in the trigeminal mesencephalic nucleus (Vme). **(A)** VGLUT2 (red) and PV (blue) double labeling showed close apposite of VGLUT2 positive terminals upon PV labeled Vme soma. The framed areas in a–d are magnified in a′–d′. Scale bar = 200 μm in a–d; 30 μm in a′–d′. **(B,C)** Western blotting analysis displayed that protein levels of VGLUT2 in the Vme of UAC cases are obviously higher than that in the control rats at different time points. **(D)** Statistical comparison of the blotting data unveiled that VGLUT2 protein levels in the UAC rats are significantly higher than that in the control cases at time points of 2–12 weeks (*p* < 0.05 at 2 weeks, *p* < 0.01 at 4–12 weeks). Protein level change is exhibited as times of fold against the control group at 3 h. *p < 0.05; **p < 0.01.

In group 2.2, double-immunofluorescent labeling for VGLUT2 and PV revealed that many VGLUT2(+) axon terminals were distributed widely around Vme neurons (Figure 5A). The increase in VGLUT2 immunoreactivity in the caudal Vme area of UAC rats was confirmed by Western blotting. Increased VGLUT2 protein levels were detected from 2 to 12 weeks post-surgery (*p* < 0.05 in the 2nd and 12th week samples; *p* < 0.01 in the 4th and 8th week preparations; Figures 5B–D) compared to those post-sham surgery.

### Attenuation of Anxiety-Related Reactions by RNA Intervention in the LHb→Vme Neuronal Pathway

As mentioned above, VGLUT2 shRNA vectors were injected in the 6th week post-surgery in UAC-treated rats. Control vehicle and UAC vehicle rats were injected in the same way without the vector. Then, the effect of VGLUT2 shRNA or vehicle only on animal behavior, VGLUT2 mRNA and protein levels in the caudal Vme, and Vme neuronal electrophysiology properties were evaluated 2 weeks after injection of the vector and vehicle.

**Figure 6:**
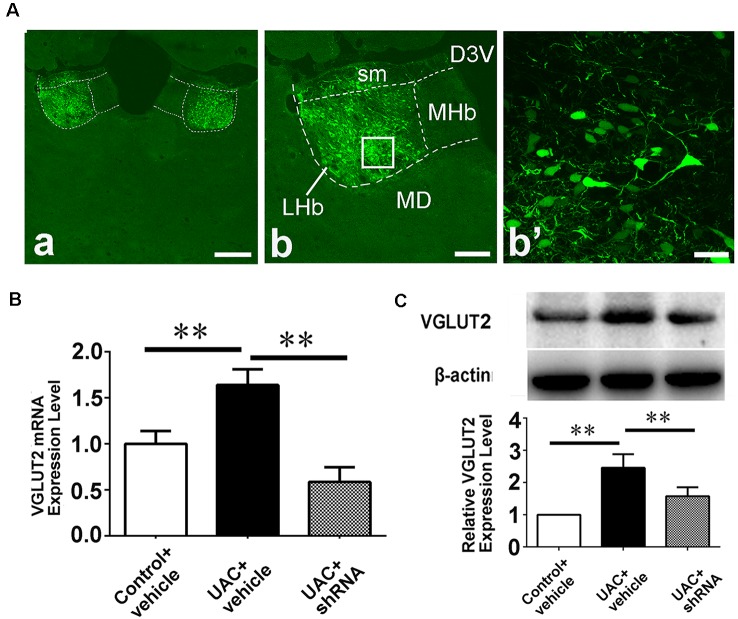
Effects of VGLUT2 shRNA vector on mRNA expression and protein levels of VGLUT2. **(A)** Integration of vectors into the LHb neurons, following injection of VGLUT2 shRNA vectors into the LHb, has been morphologically verified (a) because green fluorescent proteins are knocked in. The framed area in b is magnified in b′. Scale bar = 300 μm in a; 200 μm in b; 30 μm in b′. **(B)** Real-time PCR analysis of VGLUT2 expression revealed VGLUT2 mRNA expression was increased in the LHb neurons of UAC vehicle-treated rats compared to that of control vehicle rats (*p* < 0.01). There is a highly significant reduction of VGLUT2 mRNA expression following shRNA vector injection in UAC cases, compared to that in UAC vehicle-treated rats (*p* < 0.01). **(C)** Western blotting analysis showed that increased VGLUT2 protein level was detected in UAC vehicle rats compared to that of control vehicle rats (*p* < 0.01). Moreover, protein level of VGLUT2 in the Vme is significantly reversed by VGLUT2 shRNA vector injection, compared to that level in UAC vehicle-treated group (*p* < 0.01). **p < 0.01.

In group 3.1, the validity of the injection was evaluated through shRNA vector EGFP expression and the location of the injection site (Figure 6A). A high-magnification image of the vector injection site showed that the shRNA had been successfully transfected into LHb neurons (Figure 6A: b, b’). VGLUT2 mRNA and protein levels were compared between control vehicle, UAC vehicle, and UAC shRNA vector injection rats by using real-time PCR and Western blotting. As shown in Figures 6B,C, VGLUT2 mRNA expression was upregulated in LHb neurons of UAC vehicle-treated rats compared to that in those of control vehicle rats. VGLUT2 shRNA vector injection downregulated VGLUT2 mRNA expression in UAC shRNA vector injection rats compared to that in UAC vehicle rats (*p* < 0.01; Figure 6B). Similarly, higher VGLUT2 protein levels were detected in UAC vehicle rats than in control vehicle rats. In the Vme, shRNA injection resulted in lower VGLUT2 protein levels in UAC shRNA vector injection rats than in UAC vehicle rats (*p* < 0.01; Figure 6C).

**Figure 7:**
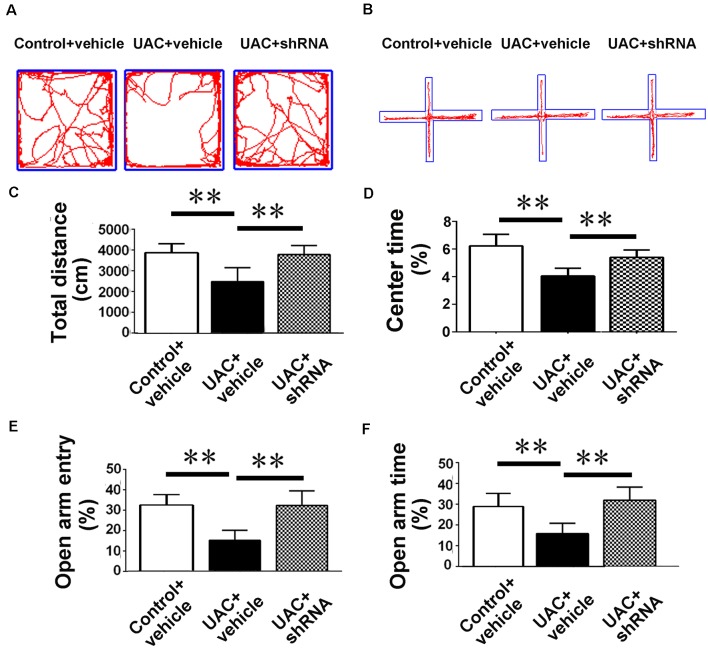
Effects of VGLUT2 shRNA vector on anxiety-related behavioral indexes. **(A,B)** Effect of VGLUT2 shRNA on anxiety-related behavioral indexes in the open field test **(A)** and elevated plus-maze test **(B)**. **(C,D)** In the open field test, UAC vehicle rats traveled shorter distance **(C)** and spent less time in the center **(D)** compared to control vehicle group. Moreover, total distance moved **(C)** and the percentage of time spent in the center area **(D)** were highly significantly improved in VGLUT2 shRNA delivered group compared with UAC vehicle group (*p* < 0.01). **(E,F)** In the elevated plus-maze test, the UAC vehicle group had a lower percentage of entries into open arms **(E)** and a lower percentage of time spent in the open arms **(F)** than the control vehicle group. Percentage of entries on open arms **(E)**, percentage of time spent in open arms **(F)** in the elevated plus-maze were highly significantly enhanced in the VGLUT2 shRNA-injected group compared with the UAC vehicle group (*p* < 0.01). **p < 0.01.

In group 3.2, both open-field testing and elevated plus-maze testing were performed 2 weeks after injections of vector and vehicle to explore whether VGLUT2 shRNA could attenuate anxiety-like behavior induced by UAC. Representative motion trails in the open-field test and elevated plus-maze test are shown in Figures 7A,B, respectively. In the open-field test, UAC vehicle rats traveled shorter distances (*p* < 0.01; Figure 7C) and spent less time in the center (*p* < 0.01; Figure 7D) than control vehicle rats did. Moreover, compared with UAC vehicle injection, shRNA injection significantly reversed the decrease in total distance and center time (*p* < 0.01; Figures 7C,D). In parallel, in the elevated plus-maze test, the UAC vehicle group had a lower percentage of entries into OAs (*p* < 0.01; Figure 7E) and a lower percentage of time spent in the OAs (*p* < 0.01; Figure 7F) than the control vehicle group did. Compared with UAC vehicle injection, shRNA injection group significantly reversed the decrease in OA time percentage (*p* < 0.01; Figure 7E) and OA entry percentage (*p* < 0.01; Figure 7F).

**Figure 8:**
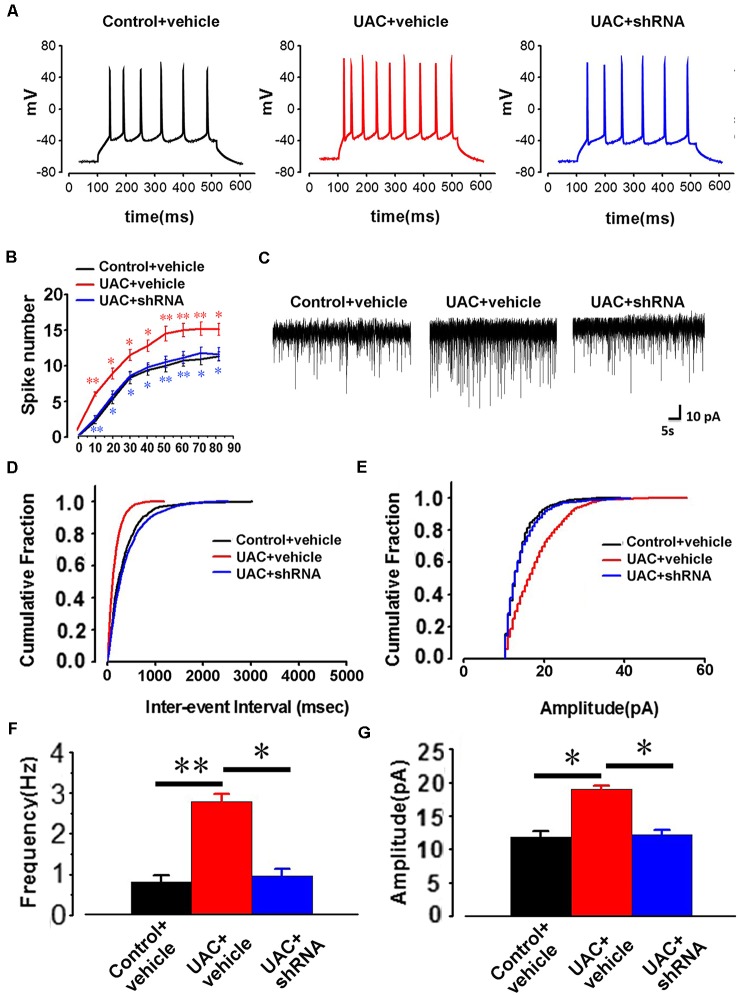
The change of AP firing frequency and sEPSCs in the trigeminal mesencephalic nucleus (Vme) neurons between control vehicle, UAC vehicle, and UAC shRNA vector injection rats. **(A)** Representative traces evoked by intracellular injection of 30-pA depolarizing currents on Vme neurons from control vehicle, UAC vehicle and UAC shRNA vector injection cases. **(B)** Average results showing that the number of APs in a train of discharges induced by the injection of step currents (400 ms, 0–80 pA) was significantly increased in UAC vehicle-treated rats. UAC increased the spike number when currents of 10 pA (*p* < 0.01), 20 pA (*p* < 0.05), 30 pA (*p* < 0.05), 40 pA (*p* < 0.05), 50 pA (*p* < 0.01), 60 pA (*p* < 0.01), 70 pA (*p* < 0.01), and 80 pA (*p* < 0.05) were applied. The shRNA injection group produced a significantly fewer number of spikes in response to currents of 10 pA (*p* < 0.01), 20 pA (*p* < 0.05), 30 pA (*p* < 0.05), 40 pA (*p* < 0.05), 50 pA (*p* < 0.01), 60 pA (*p* < 0.01), 70 pA (*p* < 0.05), and 80 pA (*p* < 0.05) compared with the UAC vehicle group. **(C)** The illustrated representative traces of sEPSCs in a Vme neuron from control vehicle, UAC vehicle, and UAC shRNA vector injection rats. **(D,E)** Cumulative fraction of the inter-event interval and amplitude of sEPSCs in the Vme neurons of control vehicle, UAC vehicle, and UAC shRNA vector injection rats. **(F,G)** Analyses of all data illustrated that the baseline frequency and amplitude of sEPSCs in UAC vehicle rats were significantly higher than those in control vehicle rats (*p* < 0.05). The frequency and amplitude were significantly lower in the shRNA-injected UAC rats (*p* < 0.05), compared to that in UAC vehicle rats. *p < 0.05; **p < 0.01.

In parallel, electrophysiological recording of 25 Vme neurons from control vehicle rats, 25 Vme neurons from UAC vehicle rats, and 35 Vme neurons from UAC shRNA vector injection rats in group 3.3 showed that Vme neuronal activity was enhanced in UAC rats. Moreover, shRNA significantly reversed the upregulation of Vme neuronal activity. Representative traces evoked by intracellular injection of 30-pA depolarizing currents on Vme neurons from control vehicle, UAC vehicle, and UAC shRNA vector injection rats are shown in Figure 8A. More averaged spikes were elicited in UAC vehicle rats than in control vehicle rats when currents of 10 pA (*p* < 0.01), 20 pA (*p* < 0.05), 30 pA (*p* < 0.05), 40 pA (*p* < 0.05), 50 pA (*p* < 0.01), 60 pA (*p* < 0.01), 70 pA (*p* < 0.01), and 80 pA (*p* < 0.05) were applied. Compared with UAC vehicle injection, shRNA injection produced significantly fewer spikes in response to currents of 10 pA (*p* < 0.01), 20 pA (*p* < 0.05), 30 pA (*p* < 0.05), 40 pA (*p* < 0.05), 50 pA (*p* < 0.01), 60 pA (*p* < 0.01), 70 pA (*p* < 0.05), and 80 pA (*p* < 0.05; Figure 8B). The baseline frequency and amplitude of sEPSCs in the UAC vehicle rats were significantly higher than those in the control vehicle rats. Moreover, compared with UAC vehicle injection, shRNA injection produced significantly lower baseline sEPSC frequency and amplitude (*p* < 0.05; Figures 8C–G).

## Discussion

**Figure 9:**
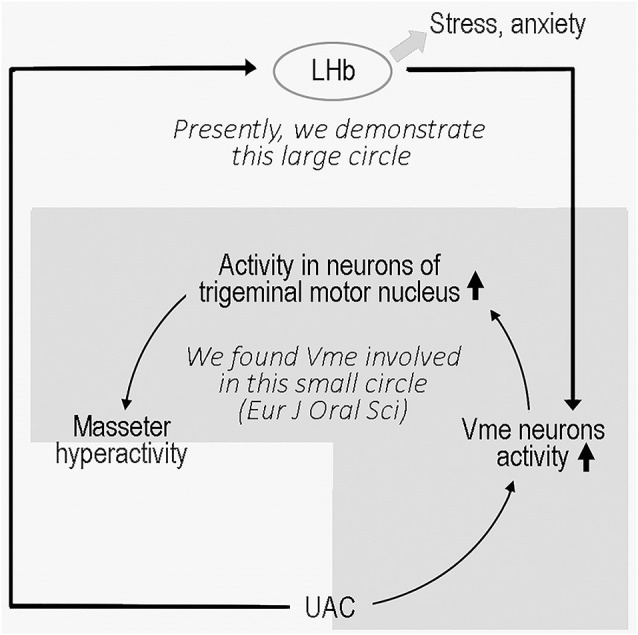
Sketch of the present story.

The LHb, located at the epithalamus, plays an important role in emotional modulation (Ullsperger and Von Cramon, 2003; Shepard et al., 2006). A previous study (Ohara et al., 2016) and our present data showed that the LHb has a direct connection with the Vme, and the previous authors verified that neurons that project to the Vme are located in the LHb area (Ohara et al., 2016) excluding the vicinity. Although there is no direct evidence that increased VGLUT2 protein in the Vme implies an increase in VGLUT2 expression in LHb neurons that project to the Vme, repression of VGLUT2 protein levels in the Vme by VGLUT2 mRNA expression in the LHb confirmed that mRNA and protein changes occurred among the same group of neurons. The electrophysiological data also implied that UAC enhanced the activities of LHb and Vme neurons along this pathway since knockdown of VGLUT2 mRNA in the LHb reversed the increased neuronal activity. In parallel, the extent of anxiety-like behaviors in UAC-modeled rats was significantly attenuated by injection of the VGLUT2-targeted shRNA AAV vector into the LHb, which downregulated both VGLUT2 mRNA in the LHb and VGLUT2 proteins in the Vme area and reduced the activity of Vme neurons. These data implied that UAC generated anxiety in modeled animals and upregulated VGLUT2 mRNA expression in the LHb. Moreover, the LHb neurons sent excitatory messages to the Vme, which provoked the Vme neurons and, according to our previous report (Liu et al., 2017), contributed to the hyperactivity of jaw closing muscles through facilitation of Vmo neuronal activity (Figure 9).

The cell bodies of the primary afferent neurons that respond to stimuli applied to the teeth have been detected in two distinct anatomical sites: the trigeminal ganglion (TG) and Vme (Byers and Dong, 1989). The primary afferents from neurons in the TG carry somatosensory information from mechanoreceptors, thermoreceptors, and nociceptors in the face, oral, and nasal cavities, while afferents from neurons in the Vme convey proprioception. In our previous study (Liu et al., 2017), we injected the cholera toxin B subunit (CTb) into the inferior alveolar nerve, which innervates the mandibular teeth. CTb-positive neurons were observed in the Vme and were considered an indication of the afferent message from the mandibular periodontal region where the proprioceptive receptors are located. In addition, CTb-positive axons were observable around neurons in the Vmo. This finding means that there is a circuit through which the neurons in the Vme receive messages from the periodontal region and deliver messages to the neurons in the Vmo.

Peripheral processes of Vme neurons conduct signals from both jaw closing muscle spindles and periodontal mechanoreceptors, and most of their central axons project to Vmo motoneurons (Pang et al., 2006). Jaw muscle spindle Vme afferents are longitudinally distributed along the whole length of the nucleus, and periodontal Vme neurons are mainly clustered in the caudal Vme (Nomura and Mizuno, 1985). Both a previous report (Ohara et al., 2016) and our own observations have shown that LHb neuron terminals are distributed along the whole column of the Vme and tracer-labeled boutons terminate on immunolabeled Vme neuronal soma therein. Hence, UAC may increase masseter hyperactivity predominantly through a neuronal circuit from periodontal Vme afferents to Vmo motoneurons, and anxiety or stress may provoke masseter overactivity through a neuronal pathway from the LHb to both the jaw muscle spindle and periodontal Vme afferents and consequently to Vmo motoneurons.

Consistently, a previous masseter electromyography recording study showed that masseter activity was increased under experimental stress and recovered to a normal level in relaxing conditions (Tsai et al., 2002). de Leeuw et al. (1994) reported that muscle dysfunction and accompanying pain were highly associated with stress-induced muscular hyperactivity. Interestingly, the authors noticed that the effect of stress on TMD depends on dental occlusion (e.g., tooth loss). Anxiety in dental patients is also associated with exacerbated trigeminal dysfunction. In addition to these case reports and studies of TMD, fear and stress promote activity in the masticatory system. For example, chewing, gnawing, and biting in rodents frequently occur in response to certain stressors (Gómez et al., 1999). In humans, diurnal tooth clenching, bruxism, and nail-biting commonly occur in subjects who feel panic (Manfredini and Lobbezoo, 2009). All of these studies suggest that the activity of the masseter muscles might be unconsciously enhanced in response to stressors. However, until the present study, which neuronal pathway connected anxiety, stress, fear, and panic to the trigeminal motor system or to tooth clenching was unclear.

However, some indirect pathways might link anxiety or stress to the hyperactivity of jaw closing muscles. For example, LHb neurons project to dorsal and median raphe nucleus serotonin neurons (Araki et al., 1988; Sego et al., 2014), which, in turn, are efferents to the Vme and Vmo (Nagase et al., 1997; Lazarov, 2002). Whether the projection from the LHb to raphe nuclei is excitatory or inhibitory remains controversial because of a lack of new neurophysiological data and stress-induced behavioral changes are not precisely associated with excitatory input from the LHb to raphe nucleus neurons (Dolzani et al., 2016). Early electrophysiological data showed that stimulation of the LHb inhibits the firing activity of serotonergic neurons in the raphe nucleus (Wang and Aghajanian, 1977). In a later report that supported this finding, LHb lesions increased serotonin levels in the dorsal raphe nucleus (Yang et al., 2008). Thus, the indirect pathway from the LHb to the Vme and Vmo might not be excitatory (Sego et al., 2014). Then, Ohara et al. (2016) found direct projections from the LHb to the Vme without exploration of its function. Nonetheless, in the present work, we showed that this direct projection facilitates Vme neuron activity, while LHb neurons are activated by stressors. This study provides the first experimental evidence linking anxiety or stress to occlusion. Furthermore, genetic intervention in LHb neuronal activity can exert an effect on this facilitation.

The limitations and implications of the present work are several: First, we showed one possibility, *via* injecting VGLUT2 shRNA into the LHb of UAC rats, that increases in VGLUT2 protein in the Vme implied an increase in VGLUT2 expression in LHb neurons that project to Vme. However, quantification of protein with a projection-specific approach seems more confirmative. Second, even though the most potent VGLUT2 shRNA vector was selected by detecting the weakest expression of mCherry in the cells, there still exists an oversight in the shRNA experiments due to non-specific effects of stereotactic injection of AAV into LHb and potential off-target effects of AAV/shRNA. Control shRNA vector should be applied to reduce the potential effects. Third, to elicit typical changes in rats, we used UAC, a specially designed rodent malocclusion. Data from animals cannot be directly transferred to humans. In human beings, posterior crossbite should be associated with chewing movement as many researchers indicated (Piancino et al., 2006, 2012). Its impact on anxiety/depression is worthy of study in the future.

Overall, our data indicated that UAC excited neurons in LHb, which mediated anxiety and exerted an excitatory impact on the Vme. Therefore, the LHb represents a potential target in patients with abnormal occlusion who are experiencing emotional stress and masseter hyperactivity, a common situation in TMD patients.

## Data Availability

The raw data supporting the conclusions of this manuscript will be made available by the authors, without undue reservation, to any qualified researcher.

## Ethics Statement

All animal studies were conducted using approved protocols and carried out in accordance with the Principles of Laboratory Animal Care (NIH Publication no.85-23, revised 1985). The Animal Care and Use Committees of the Fourth Military Medical University reviewed and approved all protocols.

## Author Contributions

XL, J-LL, and M-QW conceived and designed the experiments. XL, K-XZ, N-NY, C-KZ, M-HS, and H-YZ performed the experiments and acquired the data. XL, K-XZ, D-MW, and Z-JX analyzed the data. XL, J-DZ, J-LL, and M-QW wrote the article. All authors discussed the manuscript. All authors read and approved the final manuscript.

## Conflict of Interest Statement

The authors declare that the research was conducted in the absence of any commercial or financial relationships that could be construed as a potential conflict of interest.
